# Insights into the CD1 lipidome

**DOI:** 10.3389/fimmu.2024.1462209

**Published:** 2024-08-22

**Authors:** Rita Szoke-Kovacs, Sophie Khakoo, Peter Gogolak, Mariolina Salio

**Affiliations:** ^1^ Immunocore Ltd, Experimental Immunology, Abingdon, United Kingdom; ^2^ Department of Immunology, University of Debrecen, Debrecen, Hungary

**Keywords:** CD1, antigen presentation, self-lipid antigens, lipid mass-spectrometry, lipidome

## Abstract

CD1 isoforms are MHC class I-like molecules that present lipid-antigens to T cells and have been associated with a variety of immune responses. The lipid repertoire bound and presented by the four CD1 isoforms may be influenced by factors such as the cellular lipidome, subcellular microenvironment, and the properties of the binding pocket. In this study, by shotgun mass spectrometry, we performed a comprehensive lipidomic analysis of soluble CD1 molecules. We identified 1040 lipids, of which 293 were present in all isoforms. Comparative analysis revealed that the isoforms bind almost any cellular lipid.CD1a and CD1c closely mirrored the cellular lipidome, while CD1b and CD1d showed a preference for sphingolipids. Each CD1 isoform was found to have unique lipid species, suggesting some distinct roles in lipid presentation and immune responses. These findings contribute to our understanding of the role of CD1 system in immunity and could have implications for the development of lipid-based therapeutics.

## Introduction

The Cluster of Differentiation 1 molecules (CD1a, CD1b, CD1c, and CD1d) are Major Histocompatibility Complex (MHC) class I-like molecules that bind endogenous and exogenous lipids (1). They can activate subsets of T cells by presenting lipids on the cell surface of antigen-presenting cells. Depending on their ability to activate adaptive or innate-like T cells, CD1 molecules are divided into group 1 (CD1a, b, c) and group 2 (CD1d), respectively. A fifth molecule, CD1e, is located in the endo-lysosomes and assists lipid loading on CD1b (2). The most well-studied innate-like CD1-restricted T cells are the invariant Natural Killer T cells (iNKT), restricted to CD1d (3). Some CD1-restricted T lymphocytes recognize exogenous microbial lipids and lipopeptide antigens presented by group 1 CD1 molecules (4), while others recognize self glycosphingolipids (5, 6). Additionally, some CD1-restricted T-cell clones have been found to exhibit a dual response to both self- and microbial antigens (7). By virtue of their ability to modulate dendritic cell (DC) function, CD1-restricted T cells may play a role in both the early and late stages of the immune response (8). Recognition of microbial derived lipid antigens through CD1 molecules suggests a function of CD1-restricted T cells in anti-microbial immunity (9). Conversely, recognition of CD1-associated self-lipids is important in maintaining tissue homeostasis, and dysregulation contributes to autoimmunity (10).

Little is known about what determines the lipid repertoire captured by the different CD1 isoforms. Like classical MHC-I, CD1 molecules are synthesized in the endoplasmic reticulum, assisted by several chaperones (11, 12), and are loaded with self-lipids while they traffic to the cell surface. They are subsequently recycled through the endocytic pathway, depending on their cytoplasmic motifs (13), which might enable them to come into contact with unique lipid antigens (13). CD1a molecules primarily recycle within the early endosomes (14). This isoform has the smallest binding pocket which allows for the binding of small lipids, often from the extracellular milieu (4). CD1b molecules traffic through all endo-lysosomal compartments to the lysosomes (15, 16). CD1b has the largest binding pocket, allowing the binding of larger lipids, such as complex mycobacterial antigens (17). CD1c and CD1d both have intermediate-size binding grooves and follow similar trafficking routes, reaching the late endosomes (18, 19).

The cellular lipidome, subcellular microenvironment and the properties of the binding pocket influence the lipid repertoire bound and presented by the different CD1 isoforms. Earlier studies have characterized lipids bound to individual soluble or cleavable CD1d or CD1c molecules (20–25). Recently, using a non-targeted approach based on high-resolution mass spectrometry, Huang et al. investigated the lipidome of the four CD1 isoforms and provided a comprehensive map of self-lipid display (24, 26).

In this paper, to understand the diversity of the CD1 lipidome, we used a targeted approach, shotgun mass spectrometry, which allows the precise identification and quantification of a large number of lipids (27). Shotgun lipidomics detects the most abundant lipid contained in a sample and identifies the targeted lipids with the use of internal standards (28). In addition, this fast and highly sensitive method produces highly accurate and replicable results using minimal sample quantities (29).

We focused our investigation on soluble CD1 molecules, as the recent work by Huang et al. (26) demonstrated a significant overlap between the lipidomes eluted from recycling and non-recycling CD1a and CD1b molecules, pointing to recycling as a way to present exogenous antigens. Similar results were also observed comparing the lipidome of recycling and non-recycling murine CD1d molecules (21).

We observed that the four CD1 isoforms share a significant amount of the cellular lipidome while deviating in their preference towards certain lipid species and chain lengths. Capture of unique lipid species by each CD1 isoform implies some level of specialization in lipid presentation and specific roles during immune responses: CD1a prefers mid-sized lipids, CD1c binds a wide range of lipids including gangliosides, and CD1d uniquely captures cholesterol. These findings provide insights into lipid-antigen presentation by the CD1 molecules and help to understand how these molecules contribute to immune responses in health and disease.

## Materials and methods

### Molecular cloning and expression of CD1 molecules

The human HLA-A*02:01, CD1a (NM_001763), CD1b (NM_001764.3), CD1c (NM_001765.3) and CD1d (NM_001766.3) protein α1, α2 and α3 domains were linked to the human β2-microgobulin (β2m) via a glycine-serine linker (GGGGSGGSGSGGGSS) followed by a rigid peptide linker (PPTPSTPPT), linked C-terminal Avi-Tag™ and 6xHis tag (**Supplementary Figure 1**). They were synthesized as a single chain construct by GeneArt Gene Synthesis (Invitrogen) and subcloned into pCDNA3.1 episomal expression vector (Invitrogen), with expression driven by the IL-2 leader sequence. The pCDNA3.1 vector was engineered to co-express the BirA enzyme for biotinylation of the recombinant CD1 protein on the AviTag (**Supplementary Figure 1**). Expi293F cells (Thermo Fisher Scientific A14527) were cultivated in Expi293™ Expression Medium (Thermo FisherA1435101) and were transfected with 1µg/ml plasmid following the ExpiFectamine™ 293 Transfection Kit protocol (Life Technologies A14636). Biotinylated CD1 monomers loaded with endogenous lipids (CD1-endo) were expressed and secreted into the cell medium for five days post-transfection. They were then purified by Ni-Affinity chromatography on HisTrap excel (5ml) column (Cytiva 17371205) followed by Size Exclusion chromatography on Superdex200 increase column (Cytiva 28990944). The lipidome of the tissue culture medium is not published and we did not assess it experimentally. We cannot rule out that lipid exchange could have happened after secretion of the CD1 moleucles, however we believe high concentrations of lipids and low pH would be needed for passive ligand exchange.

### Lipid extraction for mass spectrometry lipidomics

Mass spectrometry-based lipid analysis of purified molecules was performed by Lipotype GmbH (Dresden, Germany) as described (30). Lipids were extracted using a two-step chloroform/methanol procedure (31). Gangliosides were extracted from the water phase of the preceding chloroform/methanol extraction with a solid-phase extraction protocol. Blank elution buffer was used as background control. Before extraction, samples were spiked with an internal lipid standard mixture containing: cardiolipin 14:0-14:0-14:0-14:0 (CL), ceramide 18:1;2-17:0 (Cer), diacylglycerol 17:0-17:0 (DAG), hexosylceramide 18:1;2-12:0 (HexCer), dihexosylceramide 18:1;2-12:0 (DiHexCer), Globoside 3 18:1;2-17:0 (Gb3), GM3-d3 18:1;2-18:0 (GM3), GM1-D3 18:1;2-18:0 (GM1), lyso-phosphatidate 17:0 (LPA), lyso-phosphatidylcholine 12:0 (LPC), lyso-phosphatidylethanolamine 17:1 (LPE), lyso-phosphatidylglycerol 17:1 (LPG), lyso-phosphatidylinositol 17:1 (LPI), lyso-phosphatidylserine 17:1 (LPS), phosphatidate 17:0-17:0 (PA), phosphatidylcholine 17:0-17:0 (PC), phosphatidylethanolamine 17:0-17:0 (PE), phosphatidylglycerol 17:0-17:0 (PG), phosphatidylinositol 16:0-16:0 (PI), phosphatidylserine 17:0-17:0 (PS), cholesterol ester 16:0 (CE), sphingomyelin 18:1;2-12:0;0 (SM), sulfatide 18:1;2-12:0;0 (Sulf), triacylglycerol 17:0-17:0-17:0 (TAG) and cholesterol D6 (Chol). To extract lipids from Expi293F cells, a mix of internal lipid standards was added to 1 million cells in 300µl of PBS. After extraction, the organic phase was transferred to an infusion plate and dried in a speed vacuum concentrator. The first step dry extract was re-suspended in 7.5 mM ammonium acetate in chloroform/methanol/propanol (1:2:4; V:V:V) and the second step dry extract in 33 percent ethanol solution of methylamine in chloroform/methanol (0.003:5:1; V:V:V). All liquid handling steps were performed using the Hamilton Robotics STARlet robotic platform with the Anti Droplet Control feature for organic solvent pipetting.

### Lipid identification and quantification by shotgun lipidomics

After drying and resuspension in MS acquisition mixture, lipid extracts were subjected to mass spectrometric analysis. To overcome possible MS limitations of detection due to high abundant species dominating the spectrum and reducing low abundant species with similar m/z ratio to noise, samples were analysed in replicates at high and low concentration. Two replicates of CD1 protein were analyzed in 100µg and 500µg quantities from the same protein batch. These concentrations were chosen based on an initial feasibility experiment conducted by Lipotype GmbH, where the optimal amount of 500µg of CD1 protein were identified for mass-spectrometry lipidomics. The additional 100µg replicates were analyzed to identify occurrent lipid species masked by more abundant species in the 500µg replicate. Samples were analysed by direct infusion on the QExactive hybrid quadrupole/Orbitrap mass spectrometer (Thermo Scientific) equipped with the TriVersa NanoMate automated nano-flow electrospray ion source (Advion Biosciences). All samples were analysed in both positive and negative ion modes with a resolution of R_m/z 200_ = 280000 for MS and R_m/z 200_ = 17500 for MS/MS experiments, in a single acquisition. MS/MS was triggered by an inclusion list encompassing corresponding MS mass ranges scanned in 1 Da increments (32). Both MS and MS/MS data were combined to monitor CE, Chol, DAG and TAG ions as ammonium adducts; PC and PC O-, as acetate adducts; and CL, PA, PE, PE O-, PG, PI and PS as deprotonated anions. MS only was used to monitor Gb3, Gb4, GM4, GM3, GM2, GM1, GD3, GD2, GD1, GT3, GT2, GT1, GQ1, Sulf, LPA, LPE, LPE O-, LPI and LPS as deprotonated anions, and Cer, HexCer, DiHexCer, SM, LPC and LPC O- as acetate adducts. The list of analysed lipid classes in MS and MS/MS mode can be found in supplementary data (**Supplementary Table 1**).

### Data analysis and post-processing

Data were analysed with a lipid identification software developed by Lipotype, based on LipidXplorer (33, 34). Lipid identification using LipotypeXplorer was performed on unprocessed (*.raw format) mass spectra. For MS-only mode, lipid identification was based on the molecular masses of the intact molecules. MS/MS mode included the collision-induced fragmentation of lipid molecules and lipid identification was based on both the intact masses and the masses of the fragments. Prior to normalization and further statistical analysis performed by Lipotype GmbH, lipid identified were filtered according to mass accuracy, occupation threshold, noise, and background. The lists of identified lipids and their intensities were stored in a database optimized for the particular structure inherent to lipidomic datasets. The intensity of lipid class-specific internal standards was used for lipid quantification. Data post-processing and normalization were performed at Lipotype using an in-house developed data management system. Only lipid identified with a signal-to-noise ratio >5, and a signal intensity 5-fold higher than in corresponding blank samples were considered for further data analysis. The dynamic range for cell culture samples was determined prior to analysis (30). Based on these data, limits of quantification and coefficients of variation for the different lipid classes were determined. Limits of quantification were in the lower μM to sub-μM range, depending on the lipid class. The average coefficient of variation for a complete set of quantified lipid classes was around 10-15%. Each analysis was accompanied by a set of blank samples to control for a background and a set of quality control reference samples to control for intra-run reproducibility and sample specific issues. The identified lipid molecules were quantified by normalization to a lipid class-specific internal standard.

The amounts in pmoles of individual lipid molecules (species or subspecies) of a given lipid class were summed to yield the total amount of the lipid class. The amounts of the lipid classes were normalized to the total lipid amount yielding mol% per total lipids. For lipid classes that were analysed semi-quantitatively, peak intensities were normalized to the intensity of an internal standard which did not belong to the respective lipid class (normalized intensities). Additionally, normalized intensities were further standardized to total lipid content of each sample (normalized relative abundance).

We next developed an R workflow to analyse the data received from Lipotype using R version R-4.3.2. The source code is available on GitHub at: https://github.com/ritaszokekovacs/CD1-lipidome17052024.git. The R-script has functionalities for data transformation, data exploration using unsupervised learning and lipid enrichment analysis. We performed an unsupervised clustering analysis to evaluate the structure of complex data with consideration for the connections between variables. Principal component analysis (PCA) was used for exploratory data analysis. Ggfortify (0.4.17) ggcorrplot (0.1.4.1), corrr (0.4.4), FactoMineR (2.11) and factoextra (1.0.7) packages were used for PCA analysis. We generated Venn Diagrams using the ggVennDiagram (1.5.2) package. To achieve consistent visualization, plots were generated with ggplot2 (3.5.0) and viridis (0.6.5), a colour palette robust to colour blindness and greyscale printing, whenever possible.

### Data filtering and HLA-A*02:01 background

With only 81 features, which is less than 6% of the total features detected from HLA-A*02:01 controls, we assumed that the lipids detected in the CD1 samples are likely eluted from the lipid-binding groove (**Supplementary Figure 2**).

## Results

### Analysis of the lipid profiles eluted from CD1molecules: the overlapping lipidome of CD1 isoforms

We expressed four CD1 isoforms (CD1a, CD1b, CD1c and CD1d) in mammalian, Expi293F cells as soluble molecules, a format allowing detergent-free protein purification to avoid the loss of lipid-ligands. We also expressed the classical antigen-presenting molecule HLA-A*02:01 in the same cell line and analysed the lipid content eluted from it as a negative control. HLA-A*02:01 is similar in size and possesses a similar structure as CD1 molecules. However, HLA-A*02:01 captures and presents peptide antigens instead of lipids, representing a good control for non-specific binding. After affinity and size exclusion purification of the soluble molecules from the cell supernatants, lipids were eluted and profiled by mass spectrometry as described in the methods.

**Figure 1:**
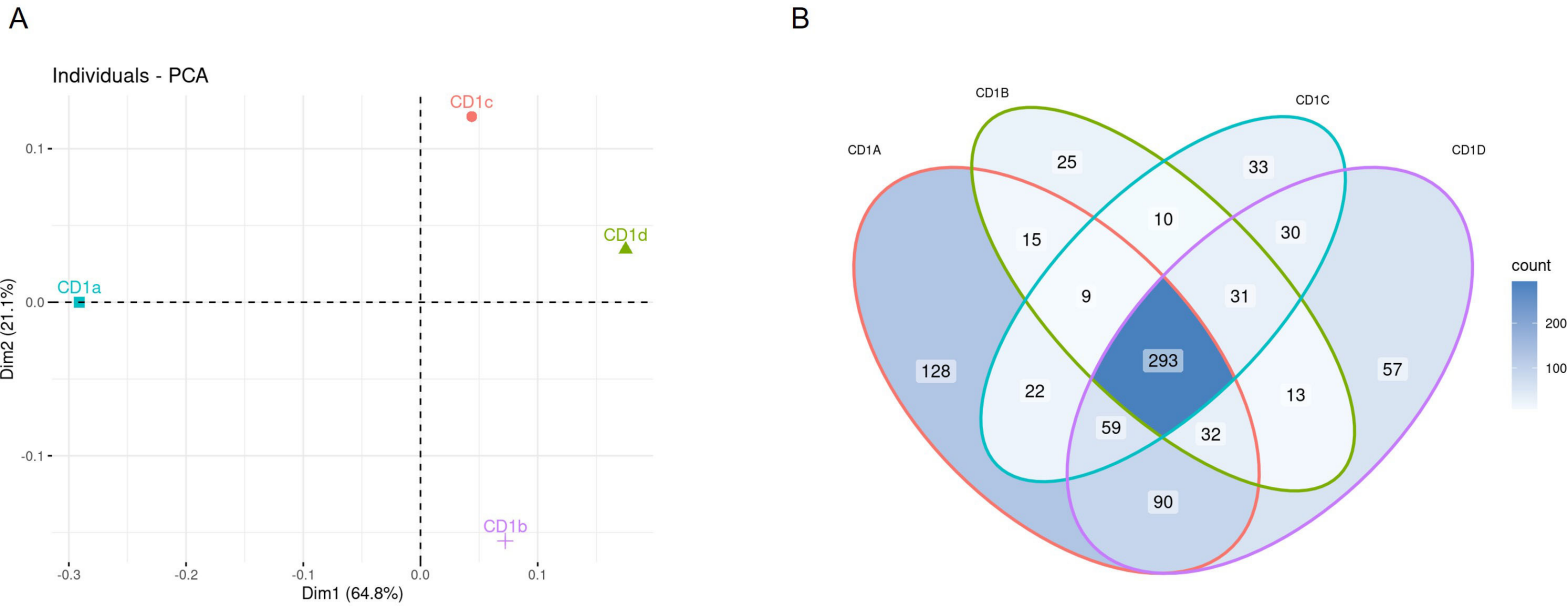
Lipidome of CD1 isoforms **(A)** Principal Component Analysis of the lipid classes bound to each CD1 isoform. **(B)** Venn diagram of overlapping lipid classes between the CD1 isoforms. The diagram shows the number events detected in each isoform.

We identified a total of 1040 lipids belonging to 39 classes, of which 29 at species level, and 10 at subspecies level in MS/MS mode (**Table 1**). As an overview of the data and to convey the most variation in the dataset, we performed a principal component analysis (PCA) on the lipid dataset, standardized to mol percentage (**Figure 1A**). We observed the largest variation in the CD1a lipidome. The lipid species that had the most influence on the principal component analysis were PC(16:0;0-18:1;0), PC(18:1;0-18:1;0), PC(16:0;0-16:1;0), SM(34:1;2), PE(18:1;0-18:1;0) and GM(34:1;2).

**Table 1:** CD1-associated ligands.

Isotype	Unique	Total
CD1a	128	648
CD1b	25	428
CD1c	33	487
CD1d	57	605

1040 lipid species were identified from the CD1 lipidome of which 293 were present in all CD1 isoforms.

The different lipid classes were non-uniformly represented across the four CD1 isoforms, with a preference of phospholipids for CD1a, gangliosides for CD1b, phosphatidylcholine for CD1c and HexCer and sphingomyelin for CD1d (**Supplementary Figure 3**). We therefore first evaluated the lipidomic overlap between the CD1 isoforms and divided the lipids into bins according to the occurrence of each feature. We observed a high lipidomic overlap between the CD1 isoforms (**Figure 1B**), suggesting broad sampling of the cellular lipidome, in agreement with recent data (26). Of the total 1040 lipids identified in the CD1 lipidome, 293 were present in all CD1 isoforms. Each isoform shared a similar level of overlap, with an average 366 shared lipids between any two isoforms; we observed the largest shared lipidome (474 species) between CD1a and CD1d molecules (**Table 1**; **Figure 1B**).

**Figure 2:**
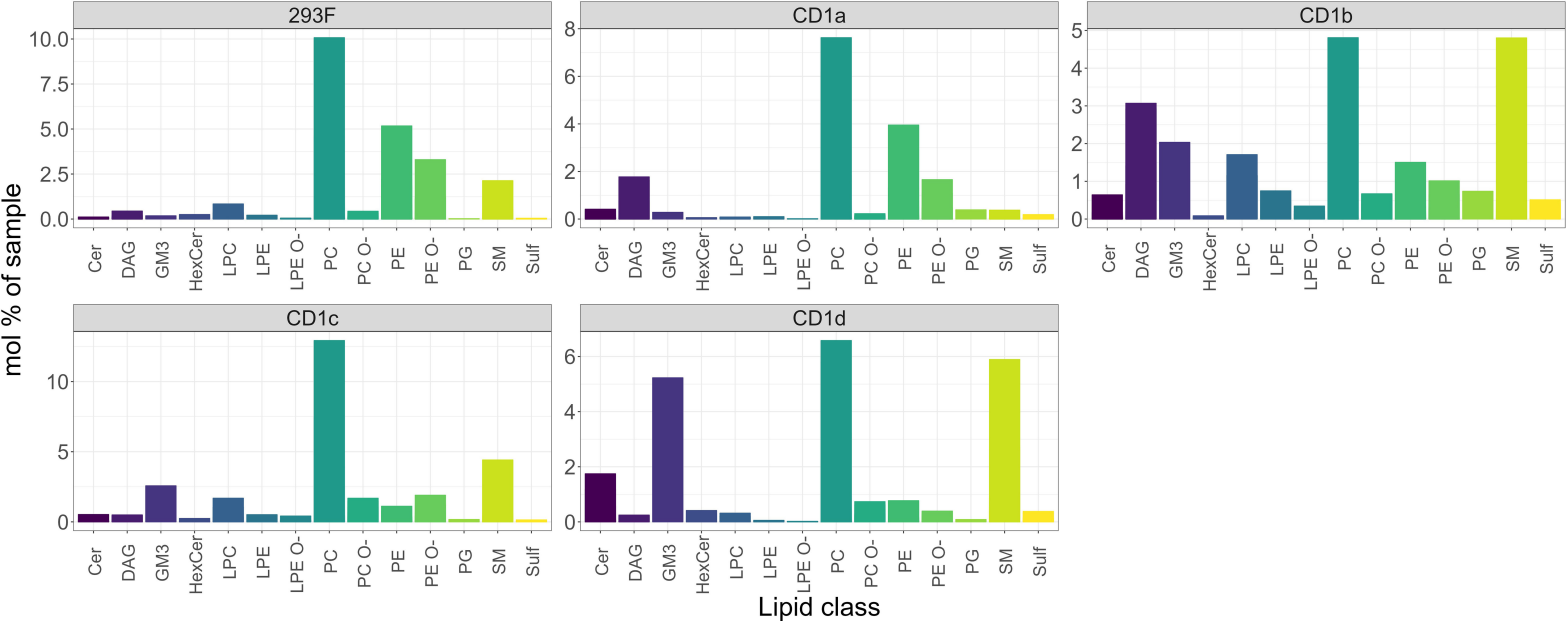
Shared cellular lipids captured by the CD1 isoforms. Mol percentage of the 293F shared lipid classes captured by the indicated CD1 isoforms in comparison with the 293F cellular lipidome. The graphs show only the proportion of lipids that are present in all isoforms. The identified lipid molecules were quantified by normalization to a lipid class-specific internal standard. The amounts in pmoles of individual lipid molecules (species of subspecies) of a given lipid class were summed to yield the total amount of the lipid class. The amounts of the lipid classes were then normalized to the total lipid amount yielding mol% per total lipids. Ceramide (Cer), Diacylglycerol (DAG), Monosialodihexosylganglioside (GM3), hexosylceramide (HexCer), lyso-phosphatidyl-cholines (LPC), ether-linked lyso-phosphatidyl-ethanolamine (LPE O-), phosphatidylcholine (PC), ether-linked Phosphatidylcholine (PC O-), phosphatidylethanolamine (PE), ether-linked phosphatidylethanolamine (PE O-), phosphatidylglycerol (PG), sphingomyelin (SM), Sulfatide (Sulf).

We next compared the classes of the 293 lipids shared between CD1 isoforms with the total cellular lipidome of Expi293F cells, and we found that – with some exceptions - the CD1 isoforms remarkably bound almost every cellular lipid species (**Figure 2**). However, while the profiles of the CD1a and CD1c lipidome were close to the cellular background, with the main enrichment found in phospholipids, the lipid profiles of CD1b and CD1d diverged: in addition to the dominant PC species, an enrichment in sphingolipid classes and sphingomyelin (SM) was also observed.

The common features present in all CD1 isoforms were 12 Ceramides (Cer), 15 diacylglycerols (DAG), 3 hexosylceramides (HexCer), 4 lyso-phosphatidyl-cholines (LPC), 4 lyso-phosphatidyl-ethanolamines (LPE), 3 ether-linked lyso-phosphatidyl-ethanolamines (LPE O-), 74 phosphatidylcholines (PC), 68 ether-linked Phosphatidylcholines (PC O-), 34 phosphatidylethanolamines (PE), 59 ether-linked phosphatidylethanolamines (PE O-), 7 sphingomyelins (SM), 4 Sulfatide species 1 phosphatidylglycerol PG (16:0;0-18:0;0) and 1 monosialodihexosylganglioside GM3 (34:1;2).

**Figure 3:**
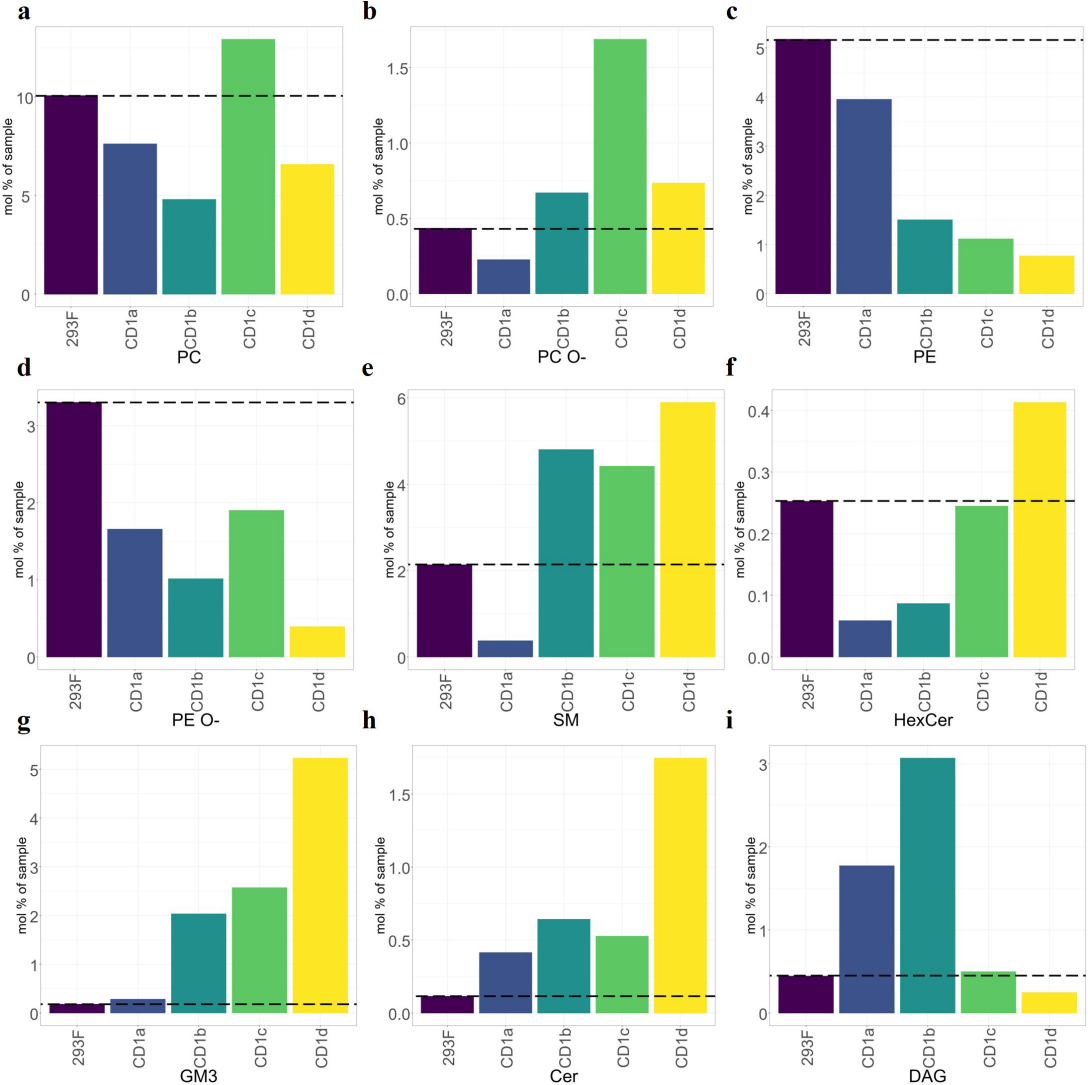
Over capture analysis of the shared CD1 lipidomes. Mol percentage of the nine most abundant shared lipid classes eluted from CD1 molecules, compared to the cellular background (dashed line). **(A-D)** Phospholipids. **(E-G)** Headed sphingolipids. **(H, I)** Headless lipids. Ceramides showed strong capture by CD1d but lower capture by other isoforms. SMs were over-captured by CD1b, CD1c and CD1d. General pattern of under-capture of phospholipids (PE, EPE, and EPC). GM3 was over-captured by CD1b, CD1c and CD1d.

To further understand the correlation between cellular abundance and capture by CD1 isoform (normalised to mol%), we restricted the analysis to the nine most abundant lipid classes (**Figure 3**). PC and ether-PC (PC O-) were present in all isoforms (**Figures 3A, B**), however, PE and ether-PE (PE O-) were under-captured by most isoforms (**Figures 3C, D**). Moderate levels of SM were captured by most isoforms, with the exception of CD1a (**Figure 3E**) (35). HexCer was only moderately over-captured by CD1d (**Figure 3F**). GM3, a headed sphingolipid was enriched 10-fold in CD1b and CD1c and 26-fold in CD1d (**Figure 3G**). Headless lipids that don’t protrude from the CD1 binding pockets like ceramides, were enriched ~2-3 fold in CD1 a, b and c and 9-fold in CD1d (**Figure 3H**). DAG was enriched 3-fold in CD1a and 6-fold in CD1b (**Figure 3I**).

### Analysis of the lipid profiles eluted from CD1 molecules: unique features detected in individual CD1 isoforms

Although we found a high level of overlap in the CD1 lipidomes, we also asked whether we could identify any isoform-specific lipid species in our dataset. To answer this question, we extracted the lipid species from the Venn diagram intersections unique for each isoform. The highest count of individual lipids was observed in the CD1a lipidome with 128 individual lipids; CD1d had 57, CD1c had 33 and CD1b contained 25 unique lipids (**Table 1**; **Figure 1B**).

**Figure 4:**
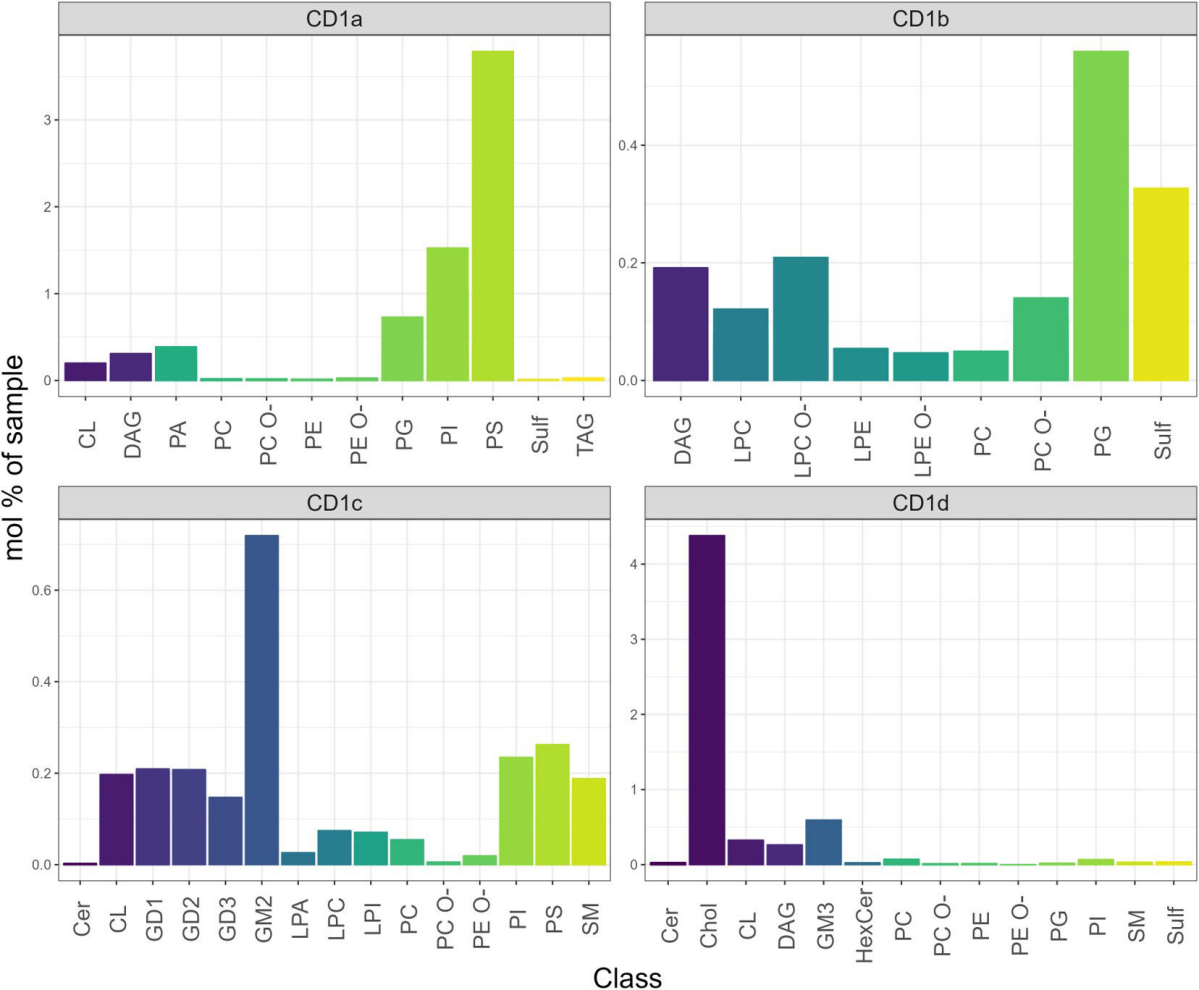
Mol% of unique lipids of the CD1 isoforms. The CD1a unique features were PI and PS; the CD1b lipidome was enriched in PG and sulfatide; the CD1c-specific lipidome was enriched in GM2 (36:1;2), GD1, and GD2 species; Cholesterol was enriched in the CD1d unique lipidome.

We observed different distributions of lipid classes amongst the unique eluate of the four CD1 isoforms (**Figure 4**; **Supplementary Figure 4**). Phosphatidylserine (18:1;0-20:2;0) (PS) was the most prominent feature amongst the CD1a-specific lipids, followed by similar chain length phosphatidylinositol (18:1;0-20:2;0) (PI) and phosphatidylglycerol (18:0;0-18:1;0) (PG). A second cluster of unique CD1a lipids comprised phosphatidic acid species (PA) as well as headless lipid species of DAG. The CD1b-specific lipid group contained 25 individual lipids, mainly lyso- and phospholipids with (18:1;0-18:1;0) and (16:1;0-18:0;0) PG and (36:1;2) and (38:0;2) sulfatide (Sulf) showing the biggest enrichment. In the CD1c-specific lipidome (33 lipids) gangliosides species GM2 (36:1;2), GD1, GD2 and GD3 were the most enriched. CD1c also uniquely accommodated shorter chain lengths (16:0;0–16:1;0) PI and PS, lysolipids (LPC, LPA, LPI and (32:2;3) and (34:2;3) sphingomyelin (SM). (**Figure 4**; **Supplementary Figure 4**).

CD1d bound to 57 unique lipids, of which cholesterol (Chol) was the most abundant. The second most abundant species bound to CD1d was the ganglioside GM3.

### Lipid chain lengths captured by CD1 isoforms

Crystal structures have shown that lipid-ligands are anchored in the CD1 molecules’ hydrophobic binding pockets (36). As the CD1 isoforms possess different structures of binding pockets (36), they can accommodate lipid antigens with acyl chains of different length and saturation. We therefore queried the acyl-chain length at the species and subspecies levels of phospholipids and glycolipids and at the species level of sphingolipids.

**Figure 5:**
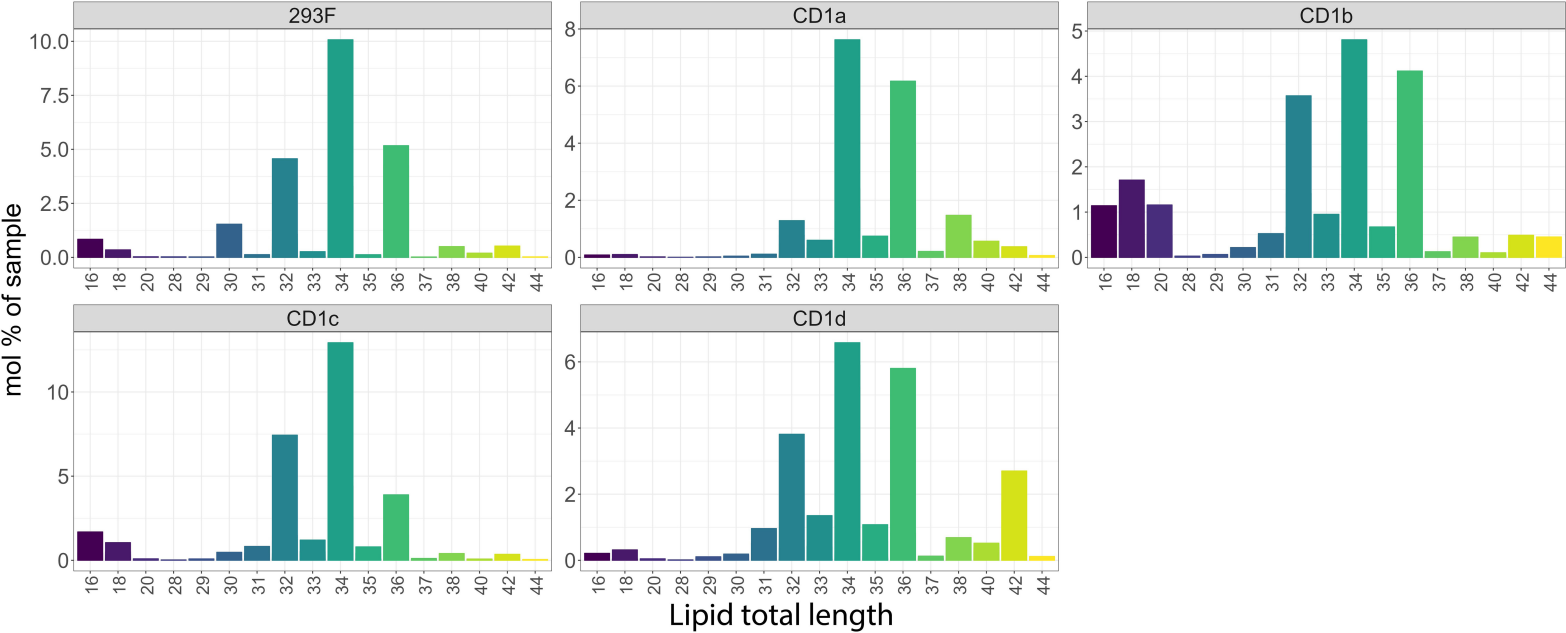
Chain length profile of the lipids captured by the CD1 isoforms. Lipid chain length captured in the shared CD1 lipidome expressed as a mol percentage of sample.

When we analysed the lipid chain length captured by the shared CD1 lipidome (**Figure 5**), we observed that CD1c molecules bound lipids similar in length to the cellular lipidome (with an enrichment for C32, C34 and C36). In the CD1a- and CD1d-specific lipid pool, we mainly found mid-sized (C34-C38) species, in agreement with previous findings (26). CD1d molecules showed an additional preference for longer C42 lipids. Compared to the cellular profile, and in agreement with previous findings (4, 17), CD1b molecules over captured short chain length lipids (C16-C20), that might be suitable spacer lipids for dual lipid presentation (37). In addition, CD1b molecules captured C32-C36 species (**Figure 5**). Short (C14-C18) lysolipids, possibly spacer lipids accommodated in the F’ pocket (36), were also found in the CD1c lipidome. Altogether, these results suggest that the binding pocket size and structure might have a unique influence in driving the ligand capture over the cellular background.

**Figure 6:**
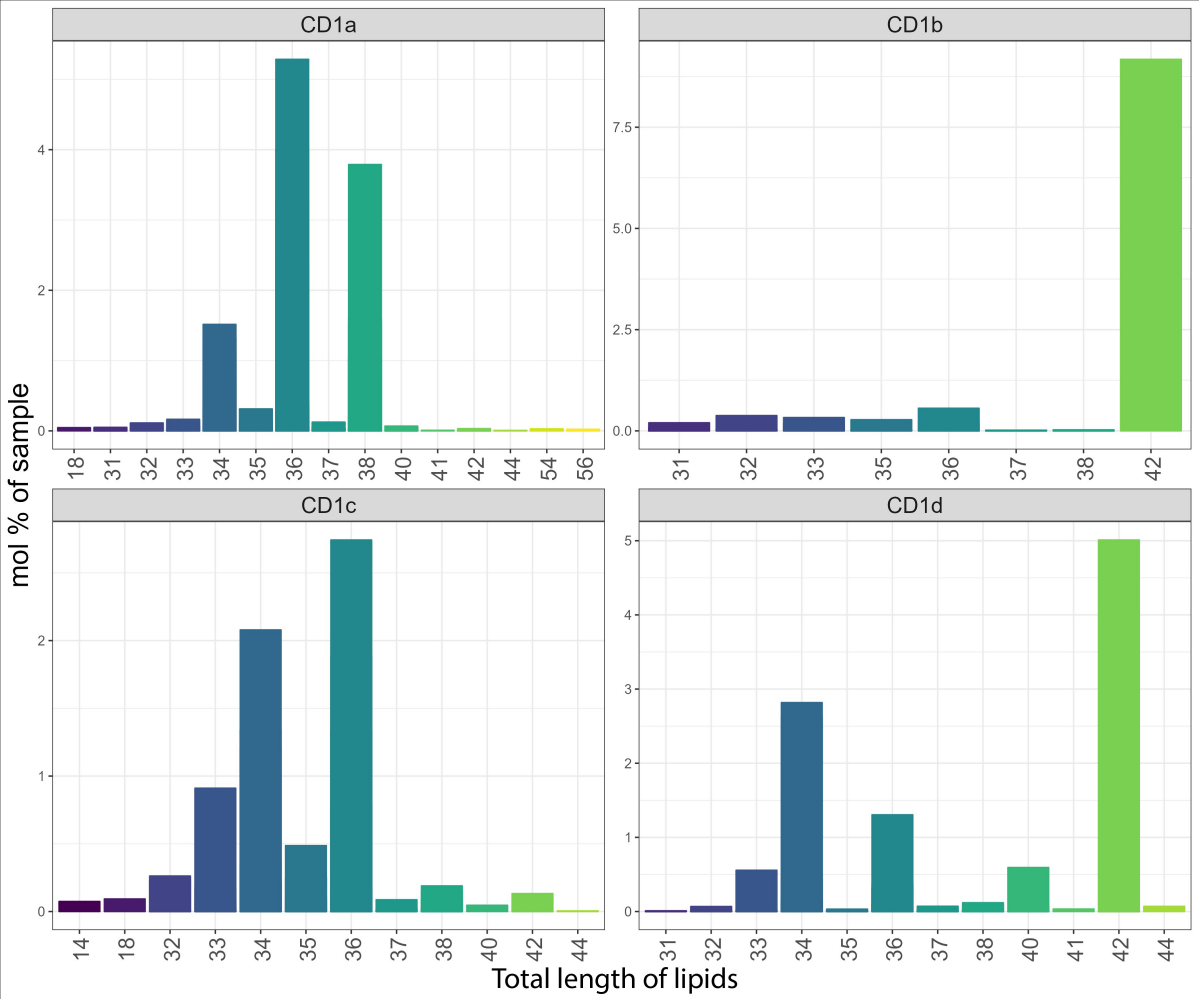
Total length of unique lipids captured by the CD1 isoforms. Lipid chain length captured in the CD1 isoform-specific lipidome expressed as a mol percentage of sample.

The chain length capture analysis of the whole lipidome was comparable to that of the shared CD1 lipidome discussed above (**Supplementary Figure 4**). In addition, larger lipids like cardiolipin (C60) and triacylglycerol (C56) were found in CD1a molecules and larger lipids (C40-C42) were captured in CD1b molecules. Large (C42) species dominated the isoform specific lipidome of CD1b and CD1d (**Figure 6**).

**Figure 7:**
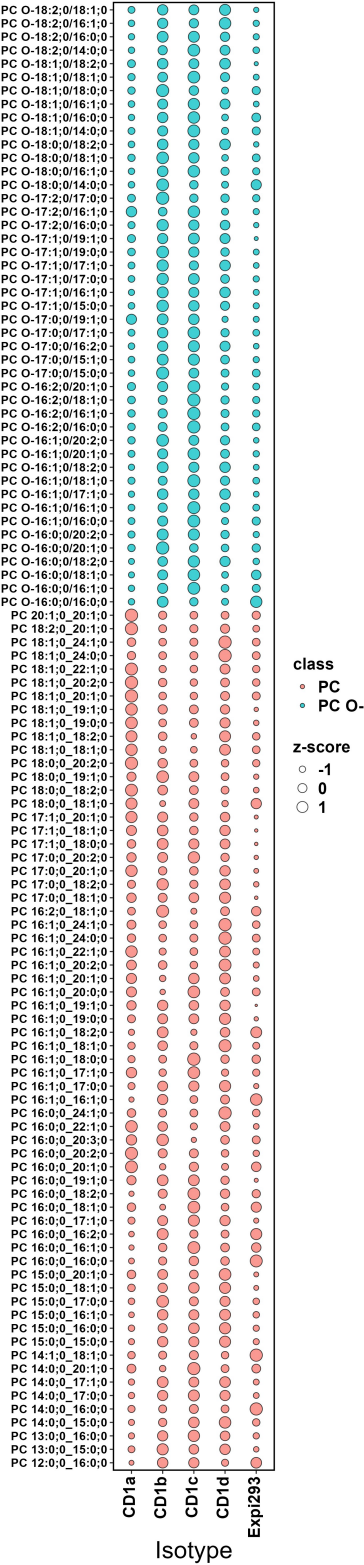
Abundance analysis of PC and PC O- species across CD1 isoforms. The bubble size indicates the abundance of the lipid species in each isoforms lipidome expressed as z-score (describing the positive or negative deviance from the mean in standard deviation units). CD1a bound PC chains of middle length (C38–C40); CD1b captured the shortest chain length of PC (C30–C38); CD1d skewed towards long-chain PCs (C42– C46); CD1c bound to the full spectrum of PC species (C30–C46).

**Figure 8:**
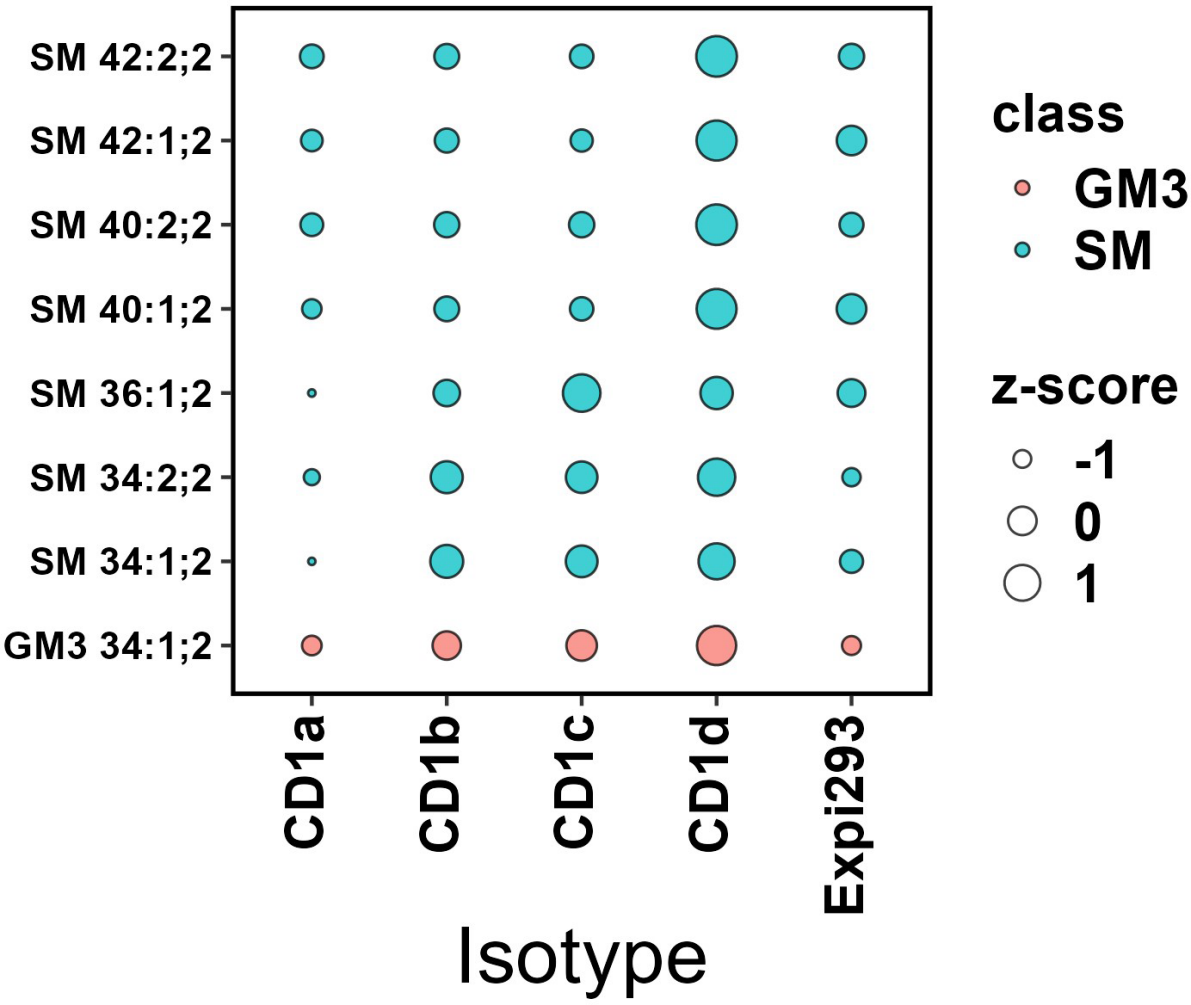
Abundance analysis of selected Sphingolipids in CD1 isoforms. The bubble size indicates the abundance of the lipid species in each isoforms’ lipidome expressed as z-score. CD1a bound to larger SM species (C40-C42); CD1b captured the shortest chain length of SMs (C34); CD1c bound to C34-C36 SMs and CD1d didn’t show any SM chain length preference. GM3 was most abundantly captured by CD1d.

We next compared the acyl-chain length distribution of the most abundant phospholipid species across CD1 isoforms. These species include PC and its ether-bound format PC O- (**Figure 7**) and SM and GM3 (**Figure 8**). We assigned z-scores to the mol% values of the lipid abundance and plotted the values on a bubble heat map. The size of the bubble on the heat map indicates the abundance of the lipid in the isoforms’ lipidome. In agreement with the above analysis, CD1a bound PC chains of middle length (C38–C40) and larger SM species (C40-C42). CD1b captured the shortest chain length of PC (C30–C38) and SMs (C34). CD1d bound long-chain PCs (C42– C46), without any preference towards SM chain length. CD1c bound to C34-C36 SMs and captured the full spectrum of PC species (C30–C46). Our data showed no preference towards specific acyl chair pairing or saturation.

## Discussion

In this study, by shotgun mass spectrometry, we performed a comprehensive analysis of the lipidome captured by soluble CD1 molecules.

The shotgun lipidomic approach only identifies lipids based on internal standards. No modified lipids nor novel targets can be identified with this method. The methods used in the recent Huang study (26) are more developed, yet we demonstrate an overlap of the two analysis platforms. Accordingly, in both datasets, the four CD1 isoforms were found to bind almost any lipids from the cell, with some lipids deviating strongly from the cellular background. We reported that the lipid profiles of CD1a and CD1c were closest to the cellular background, with the main enrichment found in phospholipids. We also observed a high level of overlap, in that 28% of the CD1 lipidome was present in all isoforms and 45-68% of the CD1 isoform’s lipidome consisted of shared lipids. However, in our dataset we also identified isoform-specific lipid species. CD1a was found to have the highest count of individual lipids (128), followed by CD1d, CD1c, and CD1b. These unique isoforms were enriched in certain lipid species, suggesting that each isoform may have a unique role in lipid presentation and subsequent immunological function.

It has been previously suggested that the small binding pocket of CD1a amongst the CD1 isoforms might limit the size of lipid-ligands captured (36). Consistent with that report, in our dataset, we observed a lipid chain length skewed towards mid-sized lipids. The lipidome eluted from the CD1a isoform closely followed the reference lipidome of Expi293F cells, which is enriched in so-called non-permissive phospholipids (PI, PS), as they have been shown to block activation of autoreactive T cell clones, with their polar head protruding from the antigen binding groove (38). In the CD1a-specific lipidome, we also identified permissive headless lipid species (DAG, CL) that might allow TCR binding via the A’ roof, in a relative lipid agnostic manner (38).

Huang and colleagues have elegantly uncovered a mismatch between size of the antigen binding groove of some CD1 molecules and chain length of the accommodated lipids (26). In our dataset, we observed that different CD1 isoforms showed a tendency towards varying lipid chain lengths: this was true at a class more than at a species level, where we did not identify preferences towards acyl chain lengths or saturation. In their study, Huang et al. demonstrated CD1 isoform specificity towards lipids with similar structures, based on lipid chain-length. Remarkably, these capture patterns were different from the order of the binding groove sizes: accordingly, we observed that both CD1b and CD1c could accommodate, in addition to C32-C36 lipids, short chain lipids (C16-C18). The presence of these small lipids in the antigen binding groove has been confirmed in several crystal structures (37, 39–41), and it has been suggested that may function as spacer lipids, preserving the fold of the CD1 molecule bound to intermediate length lipids. CD1c has a unique structure with interconnected hydrophobic channels. Fully or partially enclosed pockets and accessible portals that can accommodate structurally different lipids (36). In agreement, we observed that the lipidome eluted from the CD1c molecules mirrored the cellular lipid background and the wide spectrum of lipid chain lengths captured by this molecule suggest flexible ligand binding capabilities. Gangliosides (GD1, GD2, GD3 and GM2) were the most prominent CD1c-bound lipids, and unpublished data presented at the 2024 CD1 MR1 conference suggest they may even wrap around the alpha-helices and could aid the binding of additional lipids.

Interestingly, CD1b and CD1d diverged from the cellular lipid profile showing main enrichment in sphingolipids. These results are in agreement with findings published by Rudolph et al. (21), demonstrating that the spectrum and abundance of CD1d-associated lipids are not representative of the total cellular lipidome but rather characterized by preferential binding to long-chain sphingolipids and glycerophospholipids (21). Strikingly, CD1d was the only isoform that over-captured cholesterol. This finding is consistent with CD1d being a surface receptor for oxidized cholesterol, mediating induction, and activation of the transcription factor peroxisome proliferator-activated receptor-γ (42). Previously, cholesteryl esters had been shown to bind CD1c and stabilise its conformation to promote recognition by autoreactive T cells (39). GM3 was over captured by CD1b, CD1c and CD1d. GM3 was identified as an inhibitory natural killer T-cell ligand in 2008 (43) and may act as a modulator to fine-tune and perhaps inhibit the reactivity of Th2 NKT-cells towards self-glycolipids.

It has been demonstrated by Cotton et al. that CD1a captures endogenous sphingomyelins (C42:2), which block tetramer binding to TCRs of autoreactive T cells (35). Although we didn’t observe SM over capture by CD1a, we detected selective CD1a binding to larger SM species (C40-C42). Furthermore, in our dataset, we detected SM over capture by CD1b, c and d. By shotgun lipidomics of the CD1d associated lipidome, long chain sphingolipids, such as (SM42:2) were also detected by Rudolph et al. (21). Similarly, our data also indicated preferential binding of CD1d to longer chain SMs (C40:1,2; C40:2,2; C421,2; C42:2,2). Our recent analysis reaffirms these previously published findings, providing support for the selective binding of long chain SMs, which may represent natural endogenous T cell inhibitors (21, 44).

In conclusion, our lipidomic analysis provides valuable insights into the lipids captured by the four soluble CD1 isoforms. It highlights the unique and overlapping features of the CD1 isoforms, their preferences for certain types of lipids, and the influence of lipid chain lengths on their binding capacity. These findings contribute to our understanding of their role in immunity and could have implications for the development of lipid-based therapeutics to modulate the function of lipid reactive T cells. Indeed, lipidomic analysis represents the first step towards understanding the biological outcome of lipid antigen presentation, be it stimulatory (by so called permissive lipids), or inhibitory (presentation of non-permissive lipids, or displacement of agonist lipids). Lipidomic analysis, coupled with crystallographic studies has provided evidence for the different models of CD1-lipid recognition by T cell receptors: co-recognition of CD1 and lipid, as in the case of αβ−peptide-MHC recognition, and exemplified by CD1-αGalCer-iNKT recognition (45); absence of interference or recognition of the CD1 scaffold irrespective of the cargo, as shown for self-reactive CD1a and CD1c T cell clones (38, 46). Further lipidomic studies should encompass CD1 molecules purified from cells exposed to microbial lipids or inflamed tissues and may provide more insights into how the immune system responds to various diseases.

### Limitations of this study

While we analysed the lipidome of soluble molecules, we have not addressed the precise site of lipid loading. The lipid composition of mammalian cell organelles is not yet fully characterised, and it varies across cell types, tissues and in health versus diseased conditions. Nevertheless, it is understood that most lipids (including diacylglycerol, cholesterol, PC, PE, PI and the ceramide backbone) are synthesized in the ER and further modified (to sphingomyelin, glycosphingolipids or gangliosides) and sorted in the secretory pathway, mainly within the Golgi apparatus. This is reflected in the lipids identified in our soluble molecules, and we speculate they would be loaded in the ER and the Golgi compartments (47).

CD1 molecules were expressed as soluble proteins. These molecules do not recycle through the endo-lysosomal compartments to load lipids from these compartments. However, neither Huang and colleagues (26) nor Rudolph and colleagues (21) identified differences between the lipidome of truncated and full-length CD1 molecules.

We only expressed CD1 molecules in Expi293F cells, and we cannot exclude that molecules expressed in other cell types/states may capture additional lipid species. It would be of particular interest to characterise the lipidome of natural CD1 antigen presenting cells, such as thymocytes, dendritic cells, B cells and Langerhans cells, at steady state and in disease. These experiments are currently not possible for technical limitations.

## Data Availability

The original contributions presented in the study are publicly available. This data can be found here: https://github.com/ritaszokekovacs/CD1-lipidome17052024.
