# Supplementary material for: Insights into the CD1 lipidome
Source: Front Immunol. 2024 Aug 22;15:1462209. doi: 10.3389/fimmu.2024.1462209 (PMC11375338; doi:10.3389/fimmu.2024.1462209)
Supplement: Supplementary file 1 [file DataSheet1.docx]

Supplementary Material

# Supplementary Figures and Tables


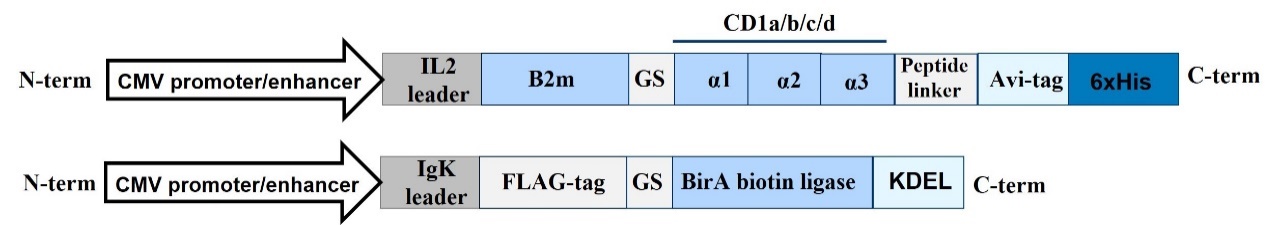


**Supplementary Figure 1.** **Schematic overview of the dual expression constructs of CD1 isoforms with BirA enzyme.**CD1 construct N term/C term: CMV promoter; ILT leader sequence; B2m macroglobulin, glycine-serine linker (GGGGSGGSGSGGGSS); CD1a/b/c/d protein α1, α2 and α3 domains, rigid peptide linker (PPTPSTPPT); Avi-TagTM; 6xHis-tag. BirA construct N term/C term: IgK leader sequence; FLAG-tag; glycine-serine linker (GGGGSGGSGSGGGSS); BirA biotin ligase, KDEL signal sequence. The BirA ligase is retained into the ER providing increased biotinylation efficiency of the produced CD1 proteins.


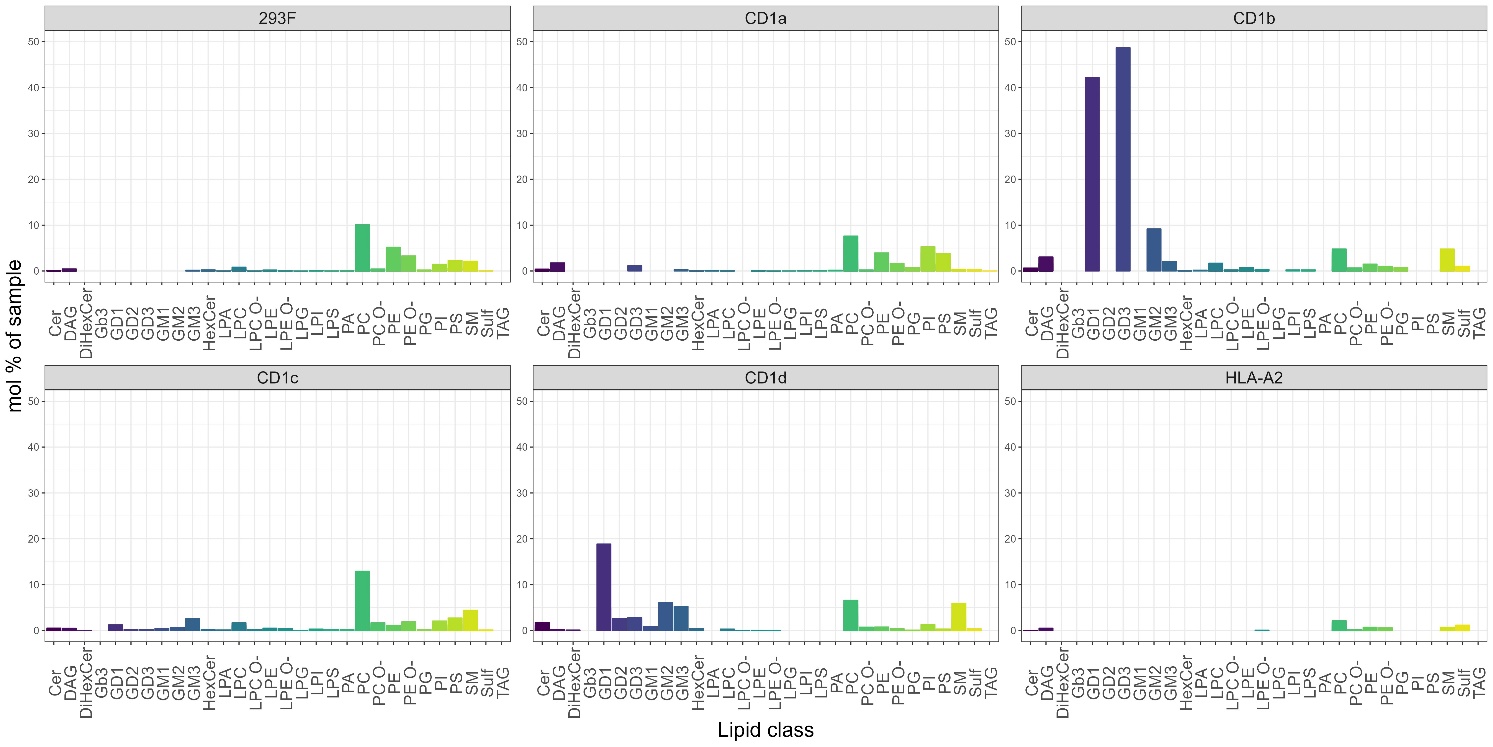


**Supplementary Figure 2.** **Mol% of lipid classes present in the lipidome of the 293F cells, CD1 isotypes and HLA-A2 control.** Less than 6% of the total features detected from HLA-A2 controls, therefore, we assumed that the lipids detected in the CD1 samples are likely eluted from the lipid-binding groove.


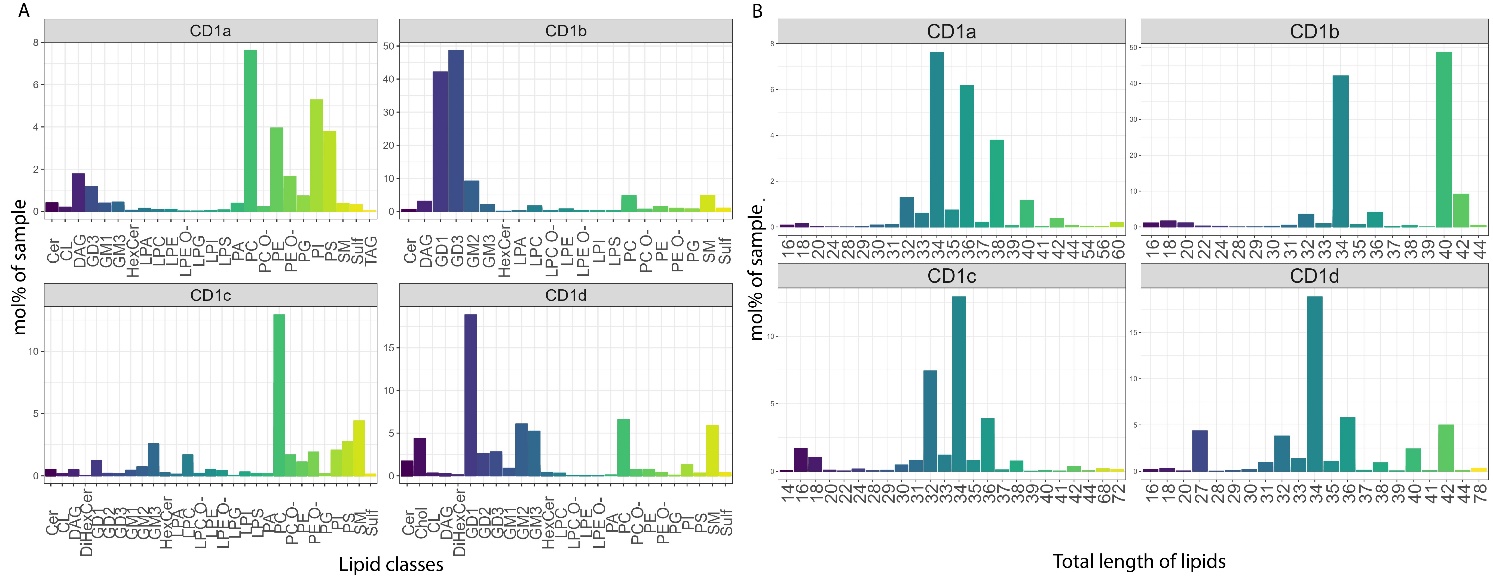


**Supplementary Figure 3.** **Cellular lipids captured by the CD1 isoforms.**(A) Lipid classes captured by the CD1 isoforms. (B) Lipid chain length of the whole cellular lipidome captured by the CD1 isoforms.


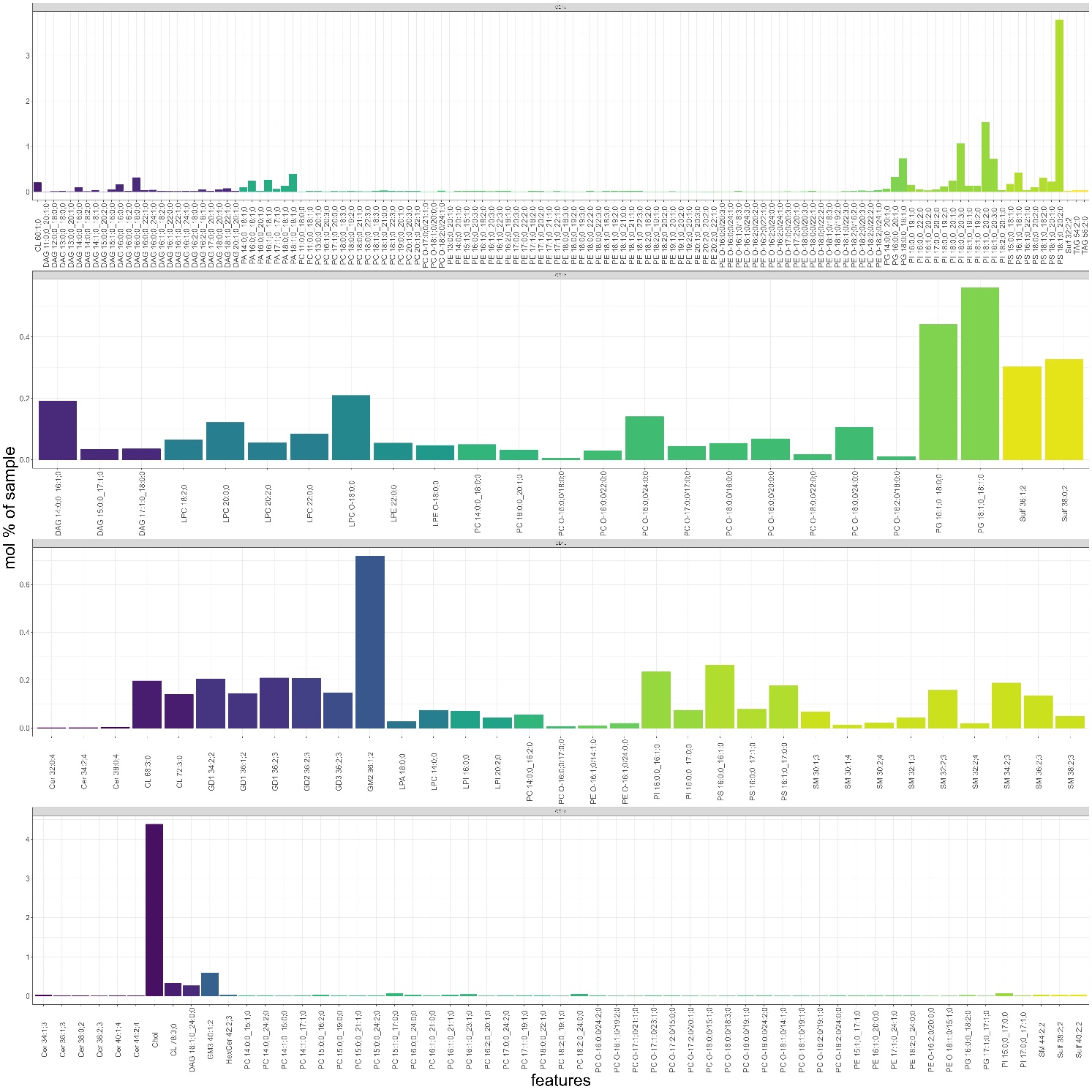


**Supplementary Figure 4.** **Mol% of unique features captured by CD1 isotypes.** (A) CD1a, (B) CD1b, (C) CD1c, (D) CD1d.

| Lipid Class | MS mode | Structural detail level | Quantification |
| --- | --- | --- | --- |
| Chol | MS | species | quantified |
| GM1 | MS | species | quantified |
| GM2 | MS | species | Semi-quantified |
| GM3 | MS | species | quantified |
| GM4 | MS | species | Semi-quantified |
| GD1 | MS | species | Semi-quantified |
| GD2 | MS | species | Semi-quantified |
| GD3 | MS | species | Semi-quantified |
| GT1 | MS | species | Semi-quantified |
| GT2 | MS | species | Semi-quantified |
| GT3 | MS | species | Semi-quantified |
| GQ1 | MS | species | Semi-quantified |
| Gb3 | MS | species | quantified |
| Gb4 | MS | species | Semi-quantified |
| Sulf | MS | species | quantified |
| DiHexCer | MS | species | quantified |
| Cer | MS | species | quantified |
| CL | MS | species | quantified |
| HexCer | MS | species | quantified |
| LPA | MS | species | quantified |
| LPC | MS | species | quantified |
| LPCO- | MS | species | quantified |
| LPE | MS | species | quantified |
| LPEO- | MS | species | quantified |
| LPG | MS | species | quantified |
| LPI | MS | species | quantified |
| LPS | MS | species | quantified |
| PA | MSMS | subspecies | quantified |
| PC | MSMS | subspecies | quantified |
| PCO- | MSMS | subspecies | quantified |
| PE | MSMS | subspecies | quantified |
| PEO- | MSMS | subspecies | quantified |
| PG | MSMS | subspecies | quantified |
| PI | MSMS | subspecies | quantified |
| PS | MSMS | subspecies | quantified |
| SM | MS | species | quantified |
| CE | MSMS | subspecies | quantified |
| DAG | MSMS | subspecies | quantified |
| TAG | MSMS | species | quantified |

**Supplementary Table 1.** List of analysed lipid classes. MS/MS mode (i.e. the fragmentation of the lipid molecules) delivers additional confidence of lipid identification and additional structural information.
